# IL-17A Secreted by Th17 Cells Is Essential for the Host against *Streptococcus agalactiae* Infections

**DOI:** 10.4014/jmb.2103.03053

**Published:** 2021-04-21

**Authors:** Jing Chen, Siyu Yang, Wanyu Li, Wei Yu, Zhaowei Fan, Mengyao Wang, Zhenyue Feng, Chunyu Tong, Baifen Song, Jinzhu Ma, Yudong Cui

**Affiliations:** 1 College of Animal Science and Veterinary Medicine, Heilongjiang Bayi Agricultural University, Daqing 163319, P.R. China; 2 College of Life Science and Technology, Heilongjiang Bayi Agricultural University, Daqing 163319, P.R. China

**Keywords:** *Streptococcus agalactiae*, T helper 17 cells, interleukin-17A

## Abstract

*Streptococcus agalactiae* is an important bacterial pathogen and causative agent of diseases including neonatal sepsis and meningitis, as well as infections in healthy adults and pregnant women. Although antibiotic treatments effectively relieve symptoms, the emergence and transmission of multidrug-resistant strains indicate the need for an effective immunotherapy. Effector T helper (Th) 17 cells are a relatively newly discovered subpopulation of helper CD4^+^ T lymphocytes, and which, by expressing interleukin (IL)-17A, play crucial roles in host defenses against a variety of pathogens, including bacteria and viruses. However, whether *S. agalactiae* infection can induce the differentiation of CD4^+^ T cells into Th17 cells, and whether IL-17A can play an effective role against *S. agalactiae* infections, are still unclear. In this study, we analyzed the responses of CD4^+^ T cells and their defensive effects after *S. agalactiae* infection. The results showed that *S. agalactiae* infection induces not only the formation of Th1 cells expressing interferon (IFN)-γ, but also the differentiation of mouse splenic CD4^+^ T cells into Th17 cells, which highly express IL-17A. In addition, the bacterial load of *S. agalactiae* was significantly increased and decreased in organs as determined by antibody neutralization and IL-17A addition experiments, respectively. The results confirmed that IL-17A is required by the host to defend against *S. agalactiae* and that it plays an important role in effectively eliminating *S. agalactiae*. Our findings therefore prompt us to adopt effective methods to regulate the expression of IL-17A as a potent strategy for the prevention and treatment of *S. agalactiae* infection.

## Introduction

*Streptococcus agalactiae*, commonly referred to as group B *Streptococcus* (GBS), frequently colonizes the gastrointestinal and genitourinary tracts of humans [1-3]. In recent years, the number of *S. agalactiae* invasive infections in human has grown, especially in neonates. Additionally, pregnant women, the elderly, cancer patients and other immunocompromised adults have high susceptibility rates [4-9]. In the affected populations, the disease spectrum ranges from mild skin and soft-tissue infections to severe sepsis, meningitis and endocarditis [10-12]. Moreover, as one of the important infectious pathogens of animals, *S. agalactiae* can also cause lots of diseases, for example, subclinical mastitis in dairy cows, which seriously impacts the animal husbandry and dairy industries [13-18]. Furthermore, it has been confirmed that the interspecies transmission of *S. agalactiae* between people and cattle is possible [19, 20]. Although *S. agalactiae* remains sensitive to β-lactam antimicrobials in general, some invasive *S. agalactiae* isolates with elevated minimum inhibitory concentrations (MICs) near the upper limit for penicillin and ampicillin have been observed across diverse geographical regions, including Canada, Japan and Kuwait [21-23]. Therefore, strategies for preventing and treating *S. agalactiae* infections have gradually shifted in the direction of immunoprevention and immunotherapy, which have received widespread attention over the past few years.

CD4^+^ T helper cells are important mediators of adaptive immune responses. Previous studies have confirmed that CD4^+^ T cells play critical roles in the host defense against *S. agalactiae* infections, which induce splenic CD4^+^ T cells from rats and mice to differentiate into Th1 cells and produce large amounts of IFN-γ [24, 25]. Moreover, the survival rate of neonatal mice with *S. agalactiae* infections is increased by the administration of recombinant IFN-γ [26].

However, after the naïve CD4^+^ T cells are activated, they can not only differentiate into effector T helper (Th) 1 cells to express IFN-γ, but may also differentiate into other types of Th cells such as Th2, Th17 and Treg [27]. By expressing different cytokines, the cells play corresponding immune functions. Th17 cells are a relatively newly discovered subpopulation of helper CD4^+^ T lymphocytes that have a unique transcriptional profile (STAT3, RORγ and RORα) and cytokine production pattern (IL-17 family), as well as specific cytokine requirements for their differentiation (TGF-β and IL-6) [28, 29]. Although the role of Th17 cells in autoimmunity is well documented, there is growing evidence that Th17 cells can recruit leukocytes (mainly neutrophils) to infection sites by producing the signature cytokine IL-17A, which plays critical roles in protecting the host from pathogenic bacterial infections [30-32].

Recent studies have shown that after many pathogenic infections, CD4^+^ T cells can be induced to differentiate into Th17 cells, and the IL-17A expression level increases. Mice lacking IL-17A and/or IL-17A receptors are highly susceptible to various pathogens, such as *Staphylococcus aureus*, *Candida albicans* and *Klebsiella pneumoniae*, which have increased colonization rates, leading to higher host mortality rates after infection. Recombinant IL-17A treatments reduce bacterial loads and increase mice survival rates [33-36]. However, whether *S. agalactiae* infection can induce the differentiation of CD4^+^ T cells into Th17 cells, and whether IL-17A can play an effective role against *S. agalactiae* infections, are still unclear.

In this study, we investigated the differentiation of Th17 cells induced by *S. agalactiae* infections and the defensive role of IL-17A against *S. agalactiae* infections. We found that *S. agalactiae* infections can induce mouse CD4^+^ T cells to differentiate into Th17 cells. Additionally, we confirmed that IL-17A plays an effective role in clearing *S. agalactiae* infections.

## Materials and Methods

### Bacterial Strains and Growth Conditions

The *S. agalactiae* HLJ-6 strain used in this study was isolated from milk samples of a dairy cow with mastitis, and the capsular polysaccharide serotype Ia type was determined by biochemical identification and multiplex PCR technology. The strain was stored in 50% glycerol at -80°C, and was preserved by the Cell Immunology Laboratory of Heilongjiang Bayi Agricultural University (China). For experiments, the isolate was cultured in brain heart infusion liquid medium (BHI, China) or on BHI agar plates at 37°C. Bacterial colony-forming units (CFUs) were quantified using serial dilution plating on BHI.

### Mice

Specific pathogen-free BALB/c mice (4-6 weeks old, females) were purchased from Changchun Institute of Biological Products (China). All the mice were housed in a specific pathogen-free facility. All the experiments were approved by the Animal Ethics Committee of Heilongjiang Bayi Agricultural University and treated in accordance with the guidelines for the care and use of laboratory animals. The approval no. for the animal experiments provided by the ethics committee is HBAU-2019002. For the experiments, 0.1 ml of the bacterial suspension or sterile vehicle solution (PBS; 0.01 M phosphate, 0.15 M NaCl, pH 7.2) was administrated intraperitoneally (i.p.).

### Proliferation Assay and Cytokine Profile Analysis of CD4^+^ T Cells

BALB/c mice were infected intraperitoneally with a dose (1×10^8^ CFU) of *S. agalactiae* (*n* =5). After 6 h, the mice were humanely sacrificed, and the spleens were harvested under aseptic conditions. A single-cell suspension was harvested by filtering spleens through a 200 μm nylon membrane. After treatment with erythrocyte-lysing buffer (Biolegend, USA), splenocytes were washed three times with RPMI-1640 (HyClone, USA) and plated at 5 × 10^6^ cells/ml in complete medium without antibiotics (RPMI-1640, 10% fetal bovine serum), and then incubated at 37°C in 5% CO_2_. After an initial 4 h incubation, the bacteriostatic agent chloramphenicol (12 μg/ml, Sigma-Aldrich, USA) was added to control the bacterial load as described previously [25]. Splenocytes from control (PBS mock-infected) animals were similarly treated (*n* = 5). After 72 h of incubation, cell proliferation was measured using a Cell Counting Kit-8 (CCK-8, Dojindo, Japan) in accordance with the manufacturer’s instructions. Briefly, 10 μl of CCK-8 was added to the culture medium, which was then incubated for an additional 4 h. The absorbance was determined at 450 nm wavelength using an ELISA Reader (Bio-Rad). The cultured cells were collected and labeled with CD4 antibody (eBioscience, USA). The proportion of CD4^+^ T cells was analyzed by flow cytometry. Cell-culture supernatants were harvested at different times (*n* = 3) and measured using commercial enzyme-linked immunosorbent assay (ELISA) kits (Dakewei, China), according to the manufacturer’s instructions.

### Isolation of Splenic Dendritic Cells (DCs) and Splenic CD4^+^ T Cells

BALB/c mice were humanely sacrificed, and then, under aseptic conditions, the spleens were harvested (*n* =3). A single-cell suspension was harvested through a 200 μm nylon mesh. After treatment with erythrocyte-lysing buffer, splenocytes were washed three times with RPMI-1640, and the cell number was determined. We used OctoMACS immunomagnetic beads (Miltenyi Biotec, Germany) to isolate CD4^+^ T cells. The CD4^+^ T-cell suspension was diluted to 5 × 10^6^ cells/ml. To further isolate the DCs, a suspension containing unlabeled cells was collected and centrifuged. The DCs were also isolated using OctoMACS immunomagnetic beads, and the DCs suspension was diluted to 1 × 10^6^ cells/ml.

### In Vitro DC-T Cell Co-Culture Model

The sorted DCs were cultured in a complete medium without antibiotics supplements, distributed in a 12-well plate at a concentration of 1 × 10^6^ cells/ml, and incubated for 1 h at 37°C in 5% CO_2_. Then, DCs were infected with *S. agalactiae* (MOI: 1) for 1 h. After a 1 h 100 μg/ml gentamycin (Sigma-Aldrich) and 5 μg/ml penicillin G (Sigma-Aldrich) treatment to kill extracellular bacteria, as described previously, DCs were washed [25]. Freshly isolated CD4^+^ T cells from naïve mice were added (5:1 CD4^+^ T cell/DC ratio). Co-cultures incubated and stimulated with PBS served as as negative controls. Cells were harvested at different time points for the flow cytometry analyses.

### Determination of the CD69 Expression Level and Percentage of Th1/Th17 Cells by Flow Cytometry

In vitro DC-T cell co-cultures were incubated for 8 h. Cells were then harvested and stained with a CD69 antibody (eBioscience), and CD69 expression was analyzed using flow cytometry. The co-cultures were incubated for 72 h, and 3 μg/ml Brefeldin A (eBioscience) was added for the last 5 h. Then, the cells were harvested, washed in RPMI-1640 and fixed with IC fixation buffer (eBioscience) for 30 min. Afterward, the cells were permeabilized in 1× permeabilization buffer (eBioscience) and incubated with fluorochrome tagged anti-IFN-γ-phycoerythrin (APC; eBioscience) and anti-IL-17A-fluorescein isothiocyanate (PE; eBioscience) for 90 min. The labeled cells were detected using flow cytometry (CytoFLEX A00-1-1102; Beckman Coulter, USA). Standard washing and incubation protocols were followed at each stage.

### Antibody Neutralization Test

In order to assess the role of IL-17A in eliminating *S. agalactiae* infection, mice were treated with commercial monoclonal antibodies (aIL-17, eBioscience) or cytokines (IL-17A, eBioscience) to block or enhance, respectively, the immune function of IL-17A in vivo. The functional grade purified aIL-17 (50 μg/100 μl) or IL-17A (1 μg/100 μl) was injected into the tail vein of BALB/c mice 24 h before *S. agalactiae* challenge or 6 h after infection (*n* = 5). Mice injected with an equal amount of PBS served as a negative control group (*n* =5). Mice were infected intraperitoneally with *S. agalactiae* (1 × 10^8^ CFUs). After 48 h, the mice organs were checked for bacterial colonization.

### Bacterial Colonization Test in the Organs

Organ samples (lung, spleen, liver and kidneys) from mice were collected under aseptic condition, and then ground and homogenized in 2 ml bacteria-free PBS. All the samples needed were diluted using the multi-proportion dilution method. A small amount of each diluted samples was cultured on BHI for 24 h at 37°C, and the number of CFUs of bacteria colonizing each organ was calculated.

### Statistical Analysis

All experiments were repeated 2-3 times. Numbers of repeats for each experiment were described in the associated figure legends. Data were expressed as the mean ± standard error of the mean (SEM) and compared using two-tailed Student’s *t*-tests. The results were analyzed using Origin Pro (v8.0; Origin Lab, USA). A *p*-value of < 0.05, < 0.01 or < 0.001 was considered statistically significant.

## Results

### Proliferation and Effector Cytokine Production by *S. agalactiae* Infection-Induced Splenic CD4^+^ T Cells

**Fig. 1:**
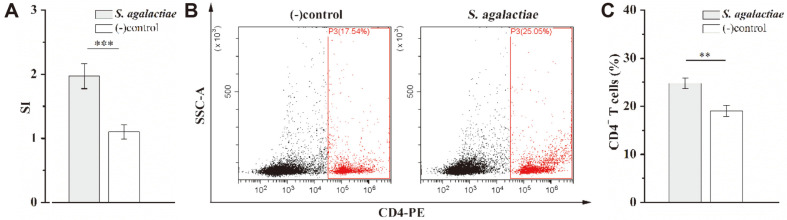
The proliferation of CD4^+^ T cells in BALB/c mice induced by *S. agalactiae*. BALB/c mice were infected intraperitoneally with a 1 × 10^8^ CFU dose of *S. agalactiae* (*n* = 5). Mice injected with PBS served as negative controls (−) (*n* = 5). After 6 h, the mice were sacrificed. Splenic lymphocytes were isolated from spleen and incubated in vitro (5 × 10^6^ cells/ml). This experiment was repeated three times with similar results. Proliferation of splenic lymphocytes was detected by CCK-8 assay (**A**). The proportion of CD4^+^ T cells in the splenic lymphocytes was analyzed by flow cytometry. Representative flow cytometry plots are from one of three independent experiments that gave similar results (**B**). Bar graphs show mean numbers ± SEM tested over three independent experiments (**C**). Significant differences are indicated by ** (*p* < 0.01) and *** (*p* < 0.001).

In order to determine whether *S. agalactiae* infection induces an increase in mouse splenic lymphocytes, cells were isolated, incubated in vitro, and their proliferation after 72 h was detected by CCK-8 assay. The *S. agalactiae*-infected mouse splenic lymphocytes proliferated significantly, while the control group, injected with PBS, did not proliferate significantly (Fig. 1A). There were statistically significant differences among the two groups (*p* < 0.001). The proportion of CD4^+^ T cells in splenic lymphocytes was analyzed by flow cytometry (Figs. 1B and 1C). There were significantly more CD4^+^ T cells in *S. agalactiae*-infected mice than in the PBS negative control group (*p* = 0.007). *S. agalactiae* was shown to induce the proliferation of mice splenic CD4^+^ T cells.

**Fig. 2:**
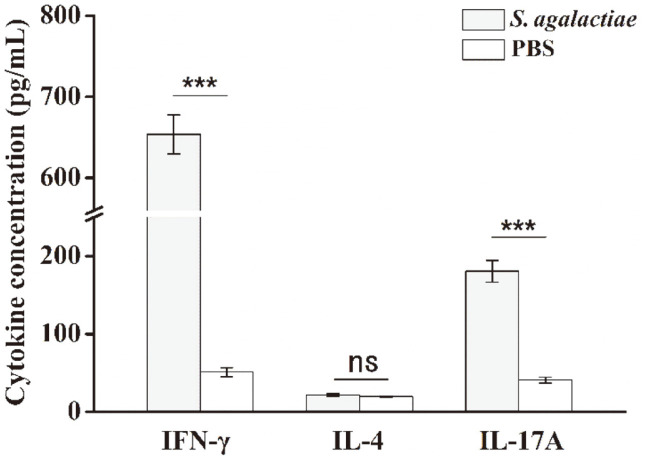
The levels of IFN-γ, IL-4 and IL-17A secreted from splenic lymphocytes were determined by ELISA. BALB/c mice were injected intraperitoneally with a 1 × 10^8^-CFU dose of *S. agalactiae* (*n* = 3). Spleens were harvested 6 h after infection and splenic lymphocytes were plated at 5 × 10^6^ cells/ml. After 4 h of incubation, the bacteriostatic agent chloramphenicol (12 μg /ml) was added. Cells were then incubated for 72 h and supernatants were collected for cytokine analysis by ELISA. Mice injected with PBS served as controls. This experiment was repeated three times with similar results. Bar graphs show mean numbers ± SEM. Significant differences are indicated by *** (*p* < 0.001) and not significant by ‘ns’.

CD4^+^ helper T lymphocytes may differentiate into specialized effector Th1 cells that secrete IFN-γ, mediating defense against intracellular microbes, or into Th2 cells that secrete IL-4, favoring IgE- and eosinophil/mast cell–mediated immune reactions against helminths, or into Th17 cells that secrete IL-17A, promoting inflammation and mediating defense against extracellular fungi and bacteria [27]. To determine the differentiation of CD4^+^ T cells induced by *S. agalactiae* infection, we collected culture supernatants and tested the expression levels of the effector cytokines IFN-γ, IL-4, and IL-17A by ELISA (Fig. 2). The splenic lymphocytes of the control group mice injected with PBS were in a normal state, and the cytokine levels that they produced were very low. The expression levels of cytokines IFN-γ and IL-17A in the splenic lymphocytes of mice infected with *S. agalactiae* showed significant increases, which in both cases were significantly greater than in the PBS control group (p <0.001). The expression level of IL-4 was lower, which was not a significant differences among the two groups (*p* = 0.06). The results showed that *S. agalactiae* infection could induce a strong CD4^+^ T cell immune response and that CD4^+^ T cells mainly differentiate into Th1 and Th17 cells, while Th2 cells were fewer.

### *S. agalactiae* Infection Induced IL-6 and TGF-β Production

**Fig. 3:**
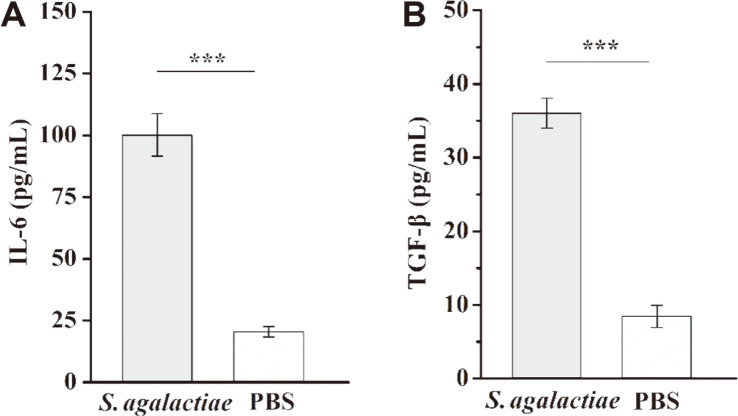
Secretion levels of IL-6 and TGF-β were determined by ELISA. A: IL-6; B: TGF-β. To determine the expression levels of IL-6 and TGF-β, BALB/c mice were infected with 1 × 10^8^-CFU dose of *S. agalactiae* (*n* = 3). Spleens were harvested 6 h after infection and splenic lymphocytes were incubated for 24 h. Supernatant was collected for measured using ELISA kits. Mice injected with PBS served as controls. This experiment was repeated three times with similar results. Bar graphs show mean numbers ± SEM. Significant differences are indicated by *** (*p* < 0.001).

After 24 h of in vitro culturing, we determined the expression levels of IL-6 and TGF-β, which regulate the responses of Th17 cells (Figs. 3A and 3B). The expression levels of IL-6 and TGF-β in *S. agalactiae*-infected mice both were significantly greater than those in the PBS control group (*p* < 0.001). These results suggested that *S. agalactiae* can induce increased expression of cytokines IL-6 and TGF-β, which regulate the response of Th17 cells. These cytokines may be involved in the differentiation of Th17 cells induced by *S. agalactiae*.

### A Major Histocompatibility Complex Class II (MHC class II) Antibody Significantly Reduced IL-17A Secretion

**Fig. 4:**
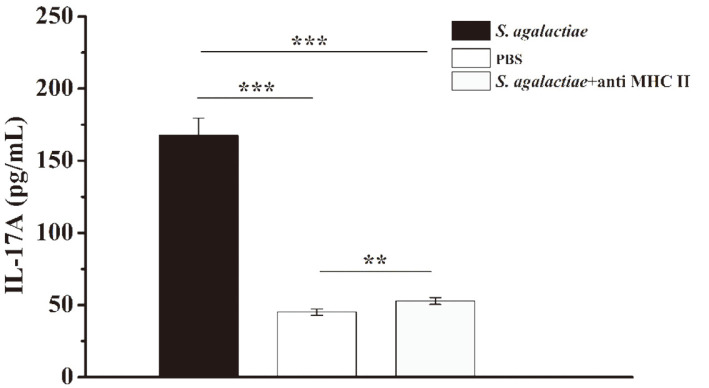
The level of IL-17A in the presence of MHC II was analyzed by ELISA. BALB/c mice were challenged with *S. agalactiae* (1 × 10^8^ CFUs/mouse) by intraperitoneal injection (*n* = 3). At 6 h post-infection, the mice were sacrificed, and their spleens were harvested. Splenic lymphocytes (5 × 10^6^ cells/mL) were isolated from spleens and cultured in the presence or absence of anti-MHC-II (10 μg/ml). The supernatants were collected, and after 72 h, the IL-17A levels were analyzed by ELISA. Mice injected with PBS served as controls (*n* = 3). This experiment was repeated three times with similar results. Bar graphs show mean numbers ± SEM. Significant differences are indicated by ** (*p* < 0.01) and *** (*p* < 0.001).

CD4^+^ T cell differentiation occurs as a result of antigen-presenting cells presenting antigen peptides to CD4^+^ T cells through MHC II molecules to promote their activation and proliferation. Consequently, we added MHC II-neutralizing antibodies during in vitro culturing to block antigen presentation. ELISA results showed that the level of IL-17A produced by the MHC II-neutralizing antibody group was extremely significantly lower than that of the experimental group without MHC II-neutralizing antibody (*p* < 0.001), but the level was still significantly higher than that of the PBS control group (*p* = 0.002) (Fig. 4). It was initially shown that the main cell source of IL-17A we detected was CD4^+^ T cells, that is, Th17 cells.

### Analyses of Percentage of *S. agalactiae*-Specific Th1 and Th17 Cells by Flow Cytometry

**Fig. 5:**
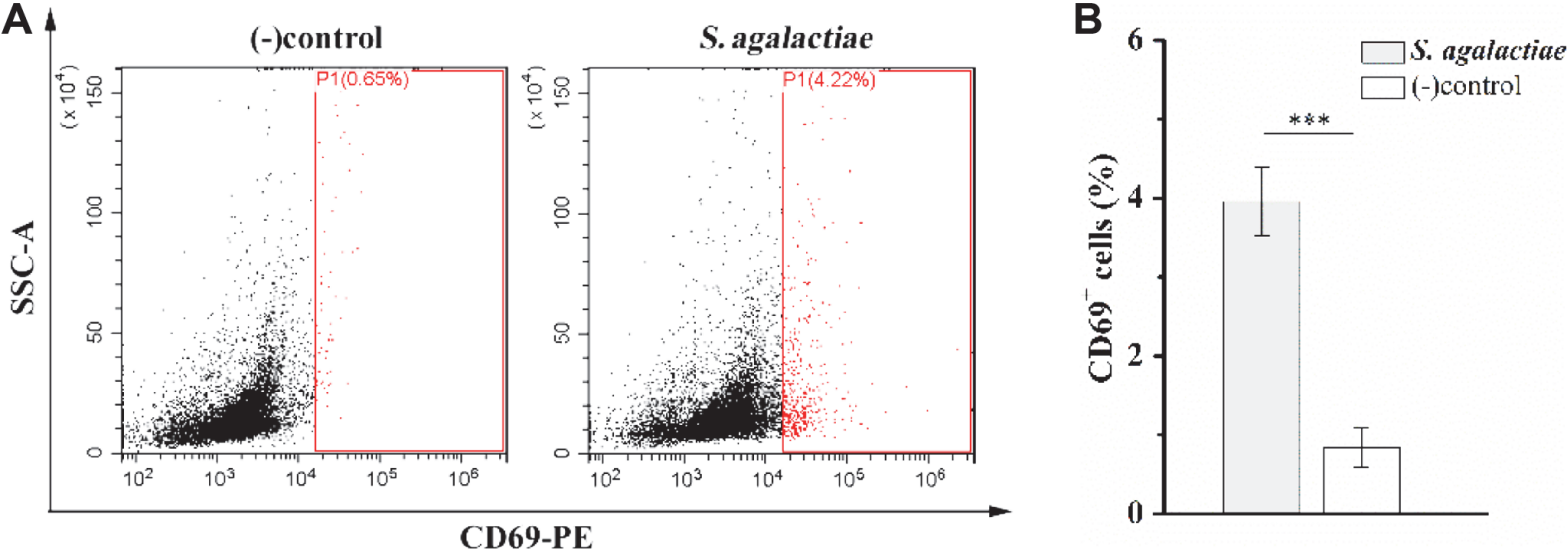
CD69 expression was analyzed by flow cytometry. DCs were infected with *S. agalactiae* for 1 h. Extracellular bacteria were killed by an antibiotic treatment and cultures were washed prior to the addition of freshly isolated splenic CD4^+^ T cells from naïve mice (5: 1 T cell: DC ratio). Co-cultures were incubated for 8 h, cells were harvested and labeled with CD69 antibody, and CD69 expression was analyzed by flow cytometry. Co-cultures incubated and stimulated with PBS served as negative controls (−). This experiment was repeated three times with similar results. Representative flow cytometry plots are from one of three independent experiments that gave similar results (**A**). Bar graphs show mean numbers ± SEM tested over three independent experiments (**B**). Significant differences are indicated by *** (*p* < 0.001).

To further exclude interference by other cells, we isolated mouse splenic CD4^+^ T cells and DC cells using Miltenyi magnetic beads, stimulated DC cells with *S. agalactiae*, and then co-cultured them with CD4^+^ T cells in vitro for 8 h. One of the earliest cell surface antigens expressed by T cells following activation is CD69 [37]. Flow cytometry showed that the CD69 molecule was expressed on the surface of CD4^+^ T cells, which was statistically significantly different from the PBS control group (*p* < 0.001) (Fig. 5). Thus, in our in vitro co-culture system, *S. agalactiae* can induce the activation of CD4^+^ T cells, confirming the effectiveness of the co-culture system.

**Fig. 6:**
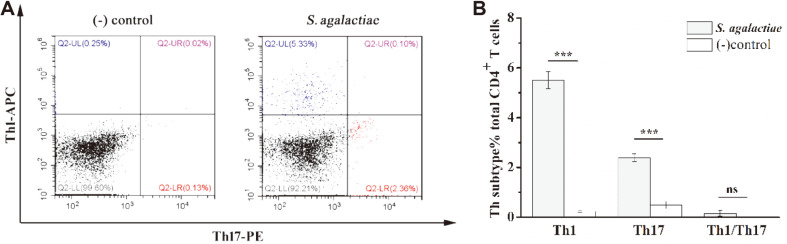
Phenotypic analysis of CD4^+^ T cells after infection with *S. agalactiae*. Co-cultures were incubated for 72 h, and Brefeldin A (3 μg/ml) was added for the last 5 h. After fixation and permeabilization, intracellular labeling was performed with anti-IFN-γ-APC and anti-IL-17A-PE. Cells were harvested and analyzed by flow cytometry. Co-cultures incubated with PBS served as negative controls (−). The experiment was repeated twice times with similar results. Representative flow cytometry plots are from one of two independent experiments that gave similar results (**A**). Bar graphs show mean numbers ± SEM tested over two independent experiments (**B**). Significant differences are indicated by *** (*p* < 0.001) and not significant by ‘ns’.

The in vitro co-culture system we established was cultured for 3 days, and a Golgi blocker was added 6 h prior to the end point. The phenotype of the CD4^+^ T cells was determined by flow cytometry (Fig. 6). A portion of the experimental group of CD4^+^ T cells produced IFN-γ, while another portion of CD4^+^ T cells produced IL-17A. When stimulated with PBS in vitro, mouse splenic CD4^+^ T cells produced very little IFN-γ or IL-17A, which was statistically significantly different in both cases from the experimental group (*p* < 0.001). There were limited IL-17/IFN-γ co-producers, which were not significant differences among the two groups (*p* = 0.06). Thus, the *S. agalactiae* infection induced a strong bacterial-specific CD4^+^ T-cell response in the spleen, which mainly consisted of Th17 cells producing IL-17A and Th1 cells producing IFN-γ.

### Role of IL-17A in Eliminating *S. agalactiae* Infection

**Fig. 7:**
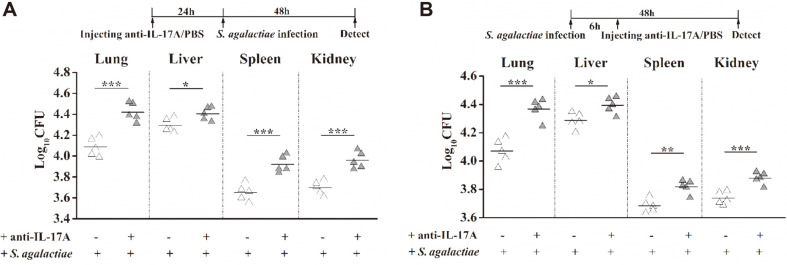
The neutralization of IL-17A resulted in an apparent increase in the bacterial colonization level in vivo. Anti-IL-17A (50 μg/100 μl) IgG was injected into the tail vein of BALB/c mice at 24 h before *S. agalactiae* challenge or 6 h after infection. Mice were injected with an equal amount of PBS as a negative control group. Mice were infected intraperitoneally with a 1 × 10^8^ CFU dose of *S. agalactiae*. At 2 days post-infection, the mice were sacrificed, and their lungs, livers, spleen and kidneys were harvested and homogenized for the determination of the CFUs in the bacterial load. Data shown are from *n* = 5 independent animals. Each symbol represents one mouse. Horizontal line indicates median. Significant differences are indicated by * (*p* < 0.05), ** (*p* < 0.01) and *** (*p* < 0.001).

To confirm the role of IL-17A in eliminating *S. agalactiae* infections, we used an in vivo antibody-neutralization experiment. At 24 h before, or 6 h after infection, mice were injected with IL-17A-neutralizing antibodies through their tail veins. The mice were infected with the maximum tolerated dose of *S. agalactiae*. At 48 h after infection, the mice were sacrificed, and the main organs were harvested for bacterial load detection. After mice were infected with *S. agalactiae*, compared with the control group injected with PBS, an injection of IL-17A-neutralizing antibodies increased the bacterial load significantly (Fig. 7). This indicated that the neutralization of IL -17A had a significant inhibitory effect on the clearance of *S. agalactiae*. Thus, the IL-17A responses appear to enhance the body’s defense capability against *S. agalactiae* infection.

**Fig. 8:**
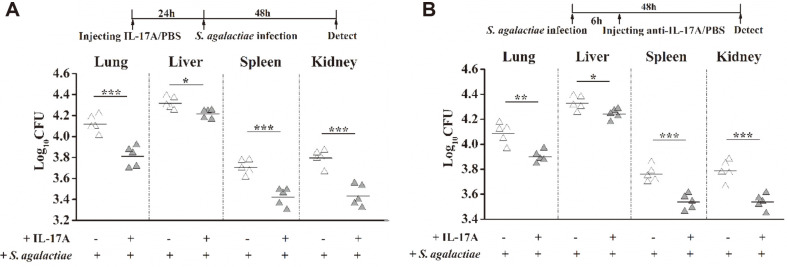
IL-17A treatments caused a significant decrease in the bacterial colonization of mouse bodies. rIL-17A (1 μg/100 μl) was injected into the tail veins of BALB/c mice at 24 h before *S. agalactiae* (1 × 10^8^ CFU) infection or 6 h after infection. Mice were injected with an equal amount of PBS as a negative control group. The method of calculating bacterial colonization of the organs was described above. Data shown are from *n* = 5 independent animals. Each symbol represents one mouse. Horizontal line indicates median. Asterisks indicate significant differences between vaccinated and control mice. Significant differences are indicated by * (*p* < 0.05), ** (*p* < 0.01) and *** (*p* < 0.001).

In order to further investigate whether the exogenous IL-17A treatment also plays a role in eliminating *S. agalactiae*, the same method was used to inject cytokine IL-17A into mice through their tail veins at 24 h before or 6 h after *S. agalactiae* infection, and the bacterial load of each major organ was detected at 48 h after infection. The bacterial loads in the liver, spleen, lung and kidneys of mice injected with exogenous IL-17A before or after infection were significantly lower than the PBS control group (Fig. 8). The result showed that the preventive treatment of IL-17A can effectively reduce the bacterial load after *S. agalactiae* infection, and the treatment at 6 h after infection can also play an effective role.

These data indicated that neutralizing endogenously produced IL-17A increases the severity of *S. agalactiae* infections. In contrast, the administration of exogenous IL-17A could effectively reduce the bacterial load in the major organs of mice. Thus, this research provides a new direction for combating *S. agalactiae* infections by regulating IL-17A responses.

## Discussion

*S. agalactiae* is a deadly pathogen that causes a variety of life-threatening infections, with neonates, pregnant women, the elderly and immune-compromised individuals, such as cancer patients, having higher susceptibility levels [1, 11, 12]. If immune-based therapy is to be an alternative to antibiotics, then a greater understanding of the protective immune responses against *S. agalactiae* infection is required.

In recent years, researchers have become more interested in how the immune responses mediated by CD4^+^ T cells help the body defend against pathogenic infections. Although the involvement of mouse CD4^+^ T cells in adaptive immunity against *S. agalactiae* infections has been studied, the previous research only focused on Th1 cells expressing IFN-γ [25]. The discovery of Th17 cells led us to re-evaluate the characteristics of CD4^+^ T-cell responses to pathogenic infections.

Numerous studies have shown that *Streptococcus pyogenes*, *K. pneumoniae*, *C. albicans* and *S. aureus* induce CD4^+^ T cells to differentiate into Th17 cells, which is accompanied by the significantly increased expression of IL-17A [33-36, 38]. IL-17A is a key player in the immune system, exhibiting roles in the defense against pathogenic infections. Thus, we speculated that *S. agalactiae* infections also induce the formation of Th17 cells, which then highly express IL-17A. To investigate this hypothesis, we first determined the expression levels of effector cytokines in mice infected with *S. agalactiae* using ELISA. The production of IFN-γ was detected in the splenic cells of infected mice, which confirmed the results of a previous study [25]. Additionally, the expression level of IL-17A was significantly increased. Because IL-17A is the main marker cytokine produced by Th17 cells, this finding suggested that Th17 cells participate in the immune response after *S. agalactiae* infections.

However, because various cells in the body express IL-17A, whether the high level of expressed IL-17A was produced by Th17 cells that differentiated from CD4^+^ T cells needed to be clarified. We confirmed, using CCK-8 assays and flow cytometry, that *S. agalactiae* infections induce the proliferation of CD4^+^ T cells, which is a prerequisite for CD4^+^ T differentiation. Cytokines are critical for the differentiation of activated antigen-specific T cells into appropriate effector T-cell lineages. TGF-β, in combination with other cytokines, directs CD4^+^ T-cell differentiation toward a Th17 phenotype. In mice, both TGF-β and IL-6 are essential for de novo Th17 differentiation [39-43]. Thus, it is necessary to evaluate whether infections induce IL-6 and TGF-β expression. We found that both IL-6 and TGF-β were highly expressed in the cell-culture supernatant, and the production of these regulatory cytokines provided the necessary conditions for CD4^+^ T cells to differentiate into Th17 cells. In addition, MHC II molecules are ligands for CD4^+^ T cells and are critical for initiating the adaptive immune response. For CD4^+^ T cells to differentiate, they must receive the antigen peptide presented by the MHC II molecule of APC [44]. When MHC II is blocked by neutralizing antibodies, the expression of IL-17A is significantly reduced. Additionally, the production is dependent on the presence of MHC II, which further confirmed that the high level of expressed IL-17A is closely related to Th17 cells.

To further exclude interference by other cells, we used Miltenyi magnetic beads to sort CD4^+^ T cells and DCs from mouse spleens, and we established an in vitro co-culture system with *S. agalactiae*. Flow cytometry detected the positive expression of CD69 molecules, which are some of the earliest cell surface antigens expressed by T cells following activation [37], indicating that the co-culture system induced CD4^+^ T cell activation. The frequencies of Th1 and Th17 cells were further analyzed using intracellular cytokine staining and flow cytometry, and both Th1 and Th17 cells were determined to be produced. This result was consistent with the cytokine ELISA results. Therefore, it confirmed our initial hypothesis that *S. agalactiae* infections induce mouse splenic CD4^+^ T cells to differentiate into Th17 cells and highly express IL-17A.

In host defensive responses, IL-17A is mostly beneficial against infections caused by extracellular bacteria, intracellular bacteria, and fungi [31, 32, 45]. Mice injected with IL-17A-neutralizing antibodies have significantly increased bacterial loads in their main organs after *S. aureus* infections [46]. The neutralization of IL-17A increases susceptibility to Chlamydia [47, 48] and greatly reduces the survival rate of mice infected with Yersinia pestis [49]. Mice lacking IL-17A showed delayed healing after *C. albicans* skin infections, and injections of exogenous IL-17A promote the faster healing of candidiasis [35]. The elimination of *Streptococcus pneumoniae* colonization is also mainly dependent on IL-17A [50].

However, uncontrolled IL-17A responses are related to a variety of human autoimmune and inflammatory diseases [30, 51]. IL-17A inhibits the protective Th1 cellular immune responses after *Aspergillus* infections [52, 53], and it is also closely related to the severity of *Helicobacter pylori* infections [54]. Thus, IL-17A does not play the same role in different infectious diseases. It enhances the body’s defenses against pathogens, but also increases the inflammatory damage to the body, which makes IL-17A functional in different pathogenic infections. Therefore, exploratory research has important scientific value.

Currently, the roles of IL-17A in defensive responses against *S. agalactiae* infections are poorly understood. This cytokine is produced in the spleen of mice infected with *S. agalactiae*. In theory, IL-17A helps clear bacteria by recruiting neutrophils to the infection site. To determine the anti-infective effects of IL-17A during of *S. agalactiae* infections, an IL-17A monoclonal antibody was injected into mice through their tail veins. After the abdominal cavity was infected with *S. agalactiae*, the bacterial load of each major organ was significantly greater than the non-neutralized group. Because the biological effects of IL-17A were blocked after IL-17A-neutralizing antibody injections, the bacterial colonization of organs after *S. agalactiae* infection was extremely significantly increased, indicating that IL-17A enhanced the ability of mice to eliminate *S. agalactiae*. Additionally, the exogenous administration of IL-17A significantly reduced the bacterial load after infection. This was confirmed by injecting cytokine IL-17A into mice and determining that the bacterial load of each major organ after *S. agalactiae* infection was significantly less than in the control group.

Previous studies have confirmed that IFN-γ secreted by Th1 cells plays an important role in host defense against *S. agalactiae* infection [24-26]. Our results further showed that *S. agalactiae* infection can also induce mouse CD4^+^ T cells to differentiate into Th17 cells expressing IL-17A, and that IL-17A is also essential for the host against *S. agalactiae* infections. Together, the data increase our understanding of the mechanisms behind *S. agalactiae* infections, help develop effective methods to regulate immune responses and can be used to aid in the prevention of *S. agalactiae* infections.
